# Successful management of severe *Pneumocystis jirovecii* pneumonia with inhaled nitric oxide and individualized ventilatory strategies in an immunosuppressed patient: a case report

**DOI:** 10.3389/fmed.2026.1808578

**Published:** 2026-05-22

**Authors:** Haobo Kong, Jingjing Pan, Jialin Liu, Min Liang, Liu Liu, Hua Niu, Ye Li

**Affiliations:** 1 Department of Respiratory Intensive Care Unit, Anhui Chest Hospital, Hefei, Anhui, China; 2 Department of Pulmonary and Critical Care Medicine, Anhui Chest Hospital, Hefei, Anhui, China; 3 Department of Tuberculosis, Anhui Chest Hospital, Hefei, Anhui, China; 4 Operating Room of Anhui Chest Hospital, Hefei, Anhui, China

**Keywords:** immunosuppression, inhaled nitric oxide, mechanical ventilation, metagenomic next-generation sequencing, *Pneumocystis jirovecii* pneumonia

## Abstract

**Background:**

Immune checkpoint inhibitors (ICIs) have improved survival in extensive-stage small-cell lung cancer (SCLC) but may cause checkpoint inhibitor pneumonitis (CIP). Management of CIP often requires prolonged high-dose corticosteroids, leading to profound immunosuppression and increased risk of opportunistic infections. Among these, *Pneumocystis jirovecii* pneumonia (PJP) is a life-threatening complication in non-HIV patients and carries higher mortality than HIV-associated PJP. Early etiological diagnosis is therefore essential. We report a case of severe PJP diagnosed by metagenomic next-generation sequencing (mNGS) and successfully managed with comprehensive respiratory support.

**Case presentation:**

A 69-year-old HIV-negative man with extensive-stage SCLC received four cycles of etoposide-platinum chemotherapy plus adebrelimab. Subsequently, CIP developed and required prolonged high-dose methylprednisolone therapy. He was transferred to our hospital for progressive dyspnea. Evaluation showed severe hypoxemia (PaO₂/FiO₂ 185 mmHg) and markedly elevated serum 1,3-*β*-D-glucan (3327.99 pg./mL). Bronchoalveolar lavage fluid mNGS identified *P. jirovecii* as the predominant pathogen, with *Klebsiella pneumoniae*, *Pseudomonas aeruginosa*, and *Candida albicans* indicating mixed pulmonary infection. The patient received trimethoprim-sulfamethoxazole, cefoperazone-sulbactam, and caspofungin. Worsening respiratory failure required endotracheal intubation and mechanical ventilation. Lung recruitment maneuvers, individualized positive end-expiratory pressure titration, and adjunctive inhaled nitric oxide progressively improved oxygenation, allowing successful extubation and eventual discharge.

**Conclusion:**

Severe PJP should be considered in non-HIV patients receiving corticosteroids for CIP. mNGS enabled rapid pathogen identification and targeted therapy. Comprehensive respiratory support, including optimized mechanical ventilation and inhaled nitric oxide, may be valuable in managing life-threatening opportunistic infections in immunosuppressed patients.

## Introduction

Immune checkpoint inhibitors (ICIs) adebrelimab combined with etoposide chemotherapy significantly improved the overall survival and progression-free survival of patients with extensive-stage small cell lung cancer (SCLC) (1). However, this therapeutic advance is accompanied by immune-related adverse events, among which checkpoint inhibitor pneumonitis (CIP) occurs in 5–10% of patients and may lead to substantial morbidity and treatment discontinuation (2). Standard management of CIP involves prolonged administration of high-dose systemic corticosteroids, which confers profound immunosuppression and substantially elevates the risk of opportunistic infections, such as PJP (3). More non-HIV (human immunodeficiency virus) PJP patients are reported as serious complications, with a mortality rate of 30–60%. Higher than HIV-related PJP patients, mainly due to delayed diagnosis and acute progression of the disease (4, 5).

In non-HIV patients, traditional diagnostic methods for PJP often lack sufficient sensitivity, while metagenomic next-generation sequencing (mNGS) of bronchoalveolar lavage fluid shows a higher detection rate and helps to quickly detect the source of infection in patients. We reported a 69-year-old male patient with SCLC who was diagnosed with CIP after PD-L1 inhibitor treatment. CIP required long-term glucocorticoid treatment and then progressed to severe mixed pulmonary infection dominated by PJP. The mNGS timely identified pathogens, early antibacterial treatment, advanced respiratory support and assisted inhalation of nitric oxide, so that patients could recover quickly and be discharged from hospital.

## Case present

A 69-year-old man was diagnosed with right lung small-cell lung cancer (SCLC) in March 2025 and subsequently received four cycles of platinum–etoposide chemotherapy combined with the PD-L1 inhibitor adebrelimab (1,200 mg). Beginning in October 2025, he gradually developed exertional dyspnea accompanied by intermittent low-grade fever. At an outside hospital, immune checkpoint inhibitor–related pneumonitis was suspected, and methylprednisolone was administered at a dose of 80 mg twice daily, in combination with empirical ceftazidime therapy of 2 g twice daily, for approximately 4 weeks. However, the patient’s respiratory symptoms did not improve, and he was transferred to our respiratory department on December 2, 2025, because of progressive dyspnea.

**Figure 1:**
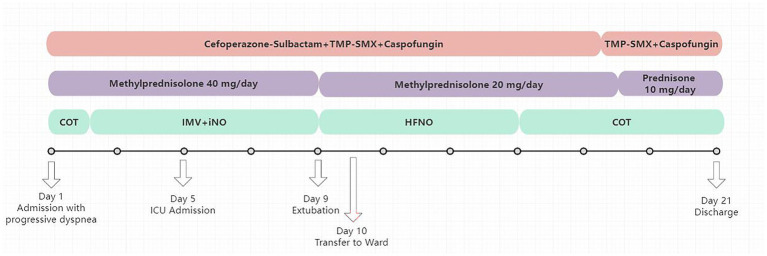
The timeline of the patient’s clinical course. ICU, Intensive Care Unit; TMP/SMZ, Trimethoprim/sulfamethoxazole; HFNO, high-flow nasal oxygen; IMV, invasive mechanical ventilation; COT, conventional oxygen therapy.

On admission, the patient was alert (Glasgow Coma Scale score, 15) and tachypneic. Vital signs were as follows: body weight 70 kg, temperature 36.5 °C, heart rate 108 beats/min, respiratory rate 26 breaths/min, blood pressure 134/77 mmHg, and oxygen saturation 85%. Laboratory tests revealed a white blood cell count of 4.31 × 10^9^/L with 94.5% neutrophils, hemoglobin 98 g/L, and platelets 141 × 10^9^/L. Inflammatory markers showed an elevated C-reactive protein level of 61.22 mg/L, while procalcitonin remained low at 0.2 ng/mL. Enzyme measurements demonstrated lactate dehydrogenase of 387 U/L and *α*-hydroxybutyrate dehydrogenase of 302 U/L. Notably, the 1,3-*β*-D-glucan assay was markedly increased at 3327.99 pg./mL. Coagulation testing demonstrated an elevated fibrinogen level of 6.3 g/L and D-dimer of 1.56 mg/L. Liver and renal function tests were within normal limits. Immunological tests: The CD4/CD8 ratio was 0.69, the absolute T lymphocyte count was 418.06 cells/μL, the absolute CD4^+^T lymphocyte count was 166.54 cells/μL, and the absolute CD8^+^ T lymphocyte count was 242.14 cells/μL. The nucleic acid test results of influenza A and B viruses were negative. Under nasal cannula oxygen therapy at 3 L/min, arterial blood gas analysis showed a pH of 7.45, PaCO₂ of 37 mmHg, PaO₂ of 61 mmHg, an oxygenation index of approximately 185 mmHg, and lactate of 1.2 mmol/L (Figure 1). After admission, bronchoscopy was performed, revealing significant congestion and edema of the bilateral bronchial mucosa with abundant viscous secretions in the lumen. Bronchoalveolar lavage was then conducted. On December 3, 2025, mNGS of bronchoalveolar lavage fluid (BALF) results indicated a positive diagnosis of *Pneumocystis jirovecii*, along with detection of *Klebsiella pneumoniae, Pseudomonas aeruginosa,* and *Candida albicans* (Table 1). BALF GM 0.1ug/L, BALF cell count indicated a neutrophil percentage of 76%. Based on the laboratory findings and the patient’s immunosuppressed status, the diagnosis was confirmed as a mixed pulmonary infection under immunosuppression, predominantly PJP. For PJP, total daily sulfamethoxazole dose of 3.84 g/day, 0.96 g every 6 h(q6h), the antimicrobial therapy was adjusted to cefoperazone-sulbactam 3.0 g intravenously, administered every 8 h. For fungal infections, capofungin was administered with an initial intravenous infusion of 70 mg on the first day, followed by 50 mg daily. Concurrently, immunoglobulin, albumin supplementation, and nutritional support therapy were provided. After admission to our hospital, intravenous infusion of methylprednisolone at 40 mg was continued to reduce the risk of immune suppression-related infection progression.

**Table 1:** mNGS results of BALF for pathogen detection.

Pathogen identification	Genus	Species	Number of sequences
bacteria	*Klebsiella*	*Klebsiella pneumoniae*	1,301
	*Pseudomonas*	*Pseudomonas aeruginosa*	428
fungus	*Pneumocystis*	*Pneumocystis jirovecii*	14,389
	*Candida*	*Candida albicans*	4,277

**Figure 2:**
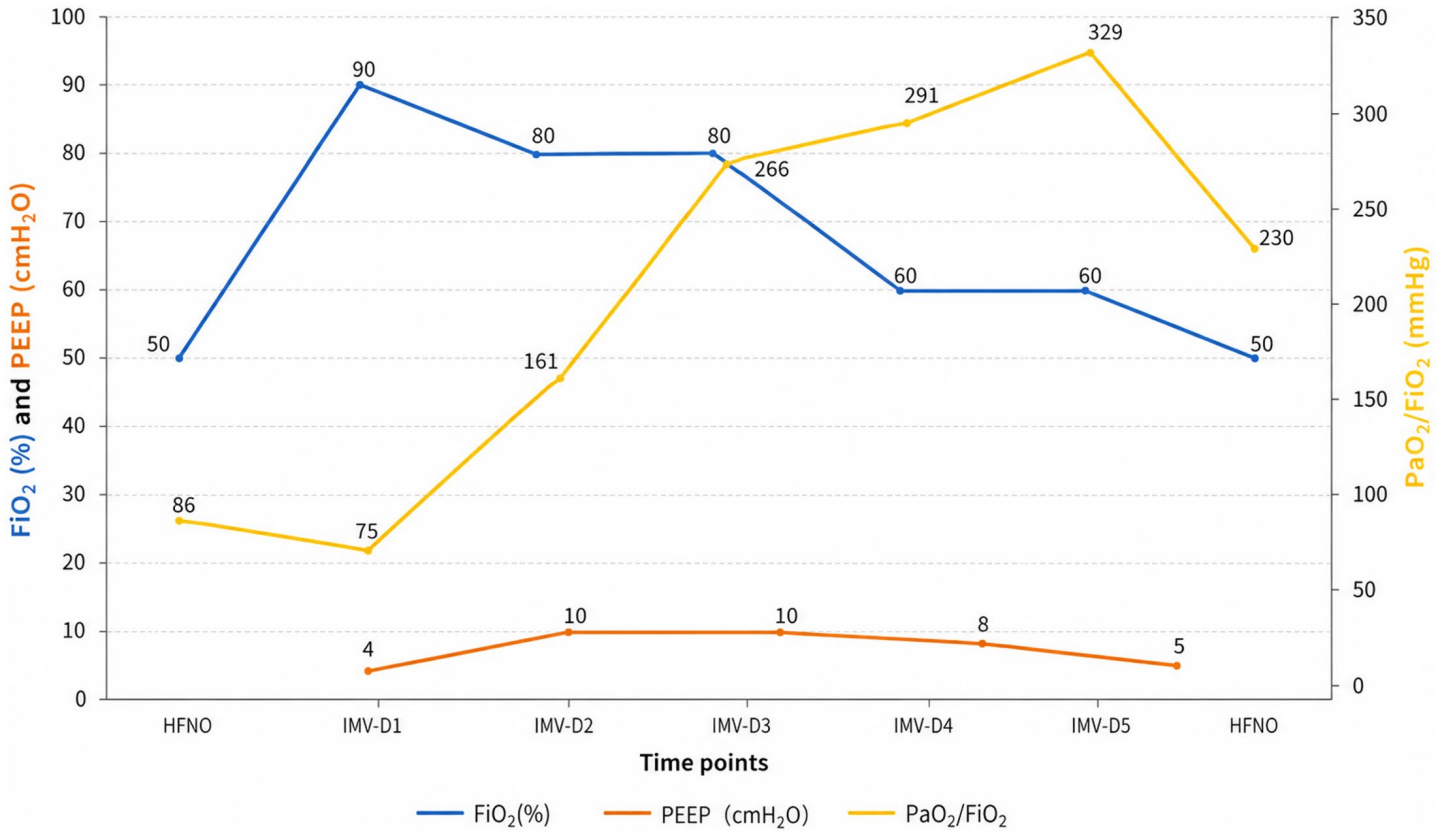
Dynamics of the patient’s respiratory therapy parameters and PaO_2_/FiO_2_. IMV: invasive mechanical ventilation; D, day; HFNO, high-flow nasal oxygen; PaO_2_, arterial partial pressure of oxygen; FiO_2_, fractional inspired oxygen; PEEP, positive end-expiratory pressure.

**Figure 3:**
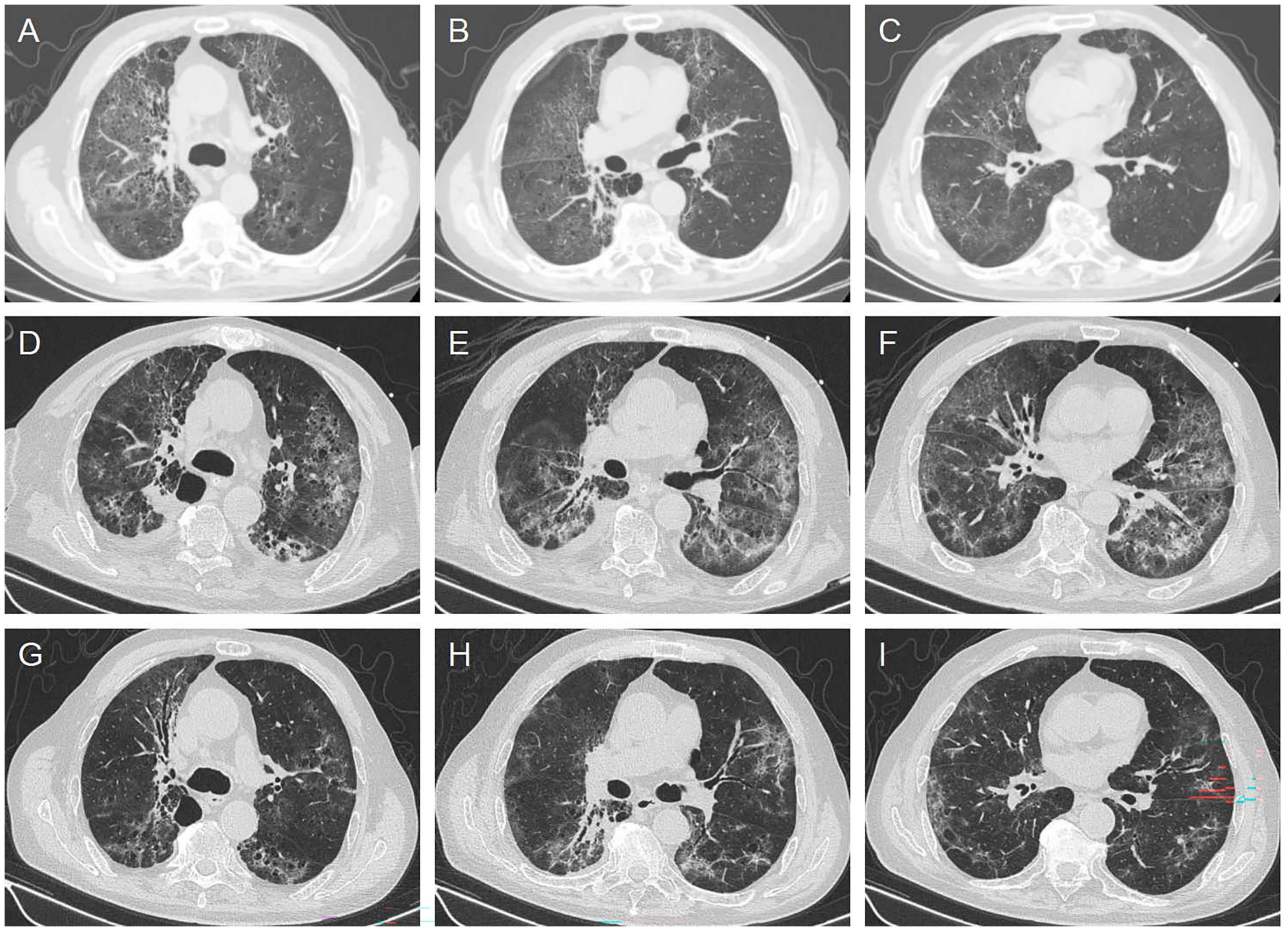
Chest computed tomography (CT) images of the patient. **(A–C)** Showed chest CT of the other hospital before admission, showing multiple ground glass shadows and interstitial changes in both lungs (2025-11-30). **(D–F)** Show CT during mechanical ventilation (2025-12-08). **(G–I)** Show the absorption of pulmonary lesions on follow-up chest CT after weaning from mechanical ventilation (2025-12-16).

Despite receiving targeted anti-infective therapy, the patient’s dyspnea continued to worsen, with progressive decline in oxygenation (Figure 2). On December 6, 2025, the patient ‘s blood oxygen saturation could not be maintained, and he was transferred to the intensive care unit for tracheal intubation and mechanical ventilation support. Initial mechanical ventilation was performed in volume-controlled mode with a tidal volume of approximately 420 mL, respiratory rate of 18 breaths per minute, FiO₂ of 60%, and PEEP of 5 cmH₂O. After deep sedation, the patient ‘s respiratory mechanics parameters were as follows: airway resistance was about 15 cmH2O/L/s, respiratory system compliance was about 41 mL/cmH2O, and endogenous positive end-expiratory pressure (PEEP) was about 2cmH2O. Based on the lung-protective ventilation strategy, perform lung re-expansion and individualized PEEP titration for the patient. The specific protocol was as follows: the upper limit of airway pressure was set at 35 cmH₂O, with an initial PEEP of 5 cmH₂O. Subsequently, PEEP was gradually increased in increments of 2 cmH₂O, with each level maintained for a 2-min plateau period. Dynamic lung compliance and oxygen saturation were continuously monitored throughout the procedure. When the airway pressure reaches the upper limit of 35 cmH₂O, the lung re-expansion is achieved by increasing the PEEP to reach this upper limit and maintaining it for 30 s. Subsequently, PEEP was gradually reduced, and the optimal PEEP level that maintained the best oxygen saturation was selected as the individualized optimal PEEP. After the above adjustment, the final ventilator parameters were set as follows: inspiratory pressure 12 cmH2O, PEEP 10 cmH2O, FiO2 80%. As an adjunctive oxygenation support measure to improve severe ventilation-perfusion mismatch, we administered inhaled nitric oxide (iNO) therapy with an initial concentration of 30 ppm. The patient’s oxygen saturation rose to 95%. In terms of anti-infection therapy, we maintained the original antibiotic regimen of the department, continuing the prescribed doses of compound sulfamethoxazole, caspofungin and cefoperazone sulbactam. The patient’s oxygenation index gradually improved. The iNO therapy was successfully discontinued after 4 days of treatment. On December 10, 2025, the patient’s oxygenation index stabilized at 250–300 mmHg, and the endotracheal intubation was successfully removed, followed by sequential nasal high-flow oxygen therapy. On December 11, the patient was transferred back to the general ward for continued treatment, with persistent improvement in respiratory status and gradual absorption of imaging lesions. By December 22,2025, the patient’s condition stabilized, and they were discharged after recovery (see Figure 3).

## Discussion

PJP secondary opportunistic infection with *P. jirovecii* is a life-threatening opportunistic infection that primarily affects patients with congenital or acquired immunodeficiency. The incidence of PJP is increasing in non-HIV immunocompromised patients, with glucocorticoid use being one of the significant risk factors, and the mortality rate in such patients is markedly higher than that in HIV-infected individuals (6). Existing studies have clearly demonstrated that long-term or high-dose glucocorticoid therapy is one of the key risk factors for the development of PJP in non-HIV populations, with the risk increasing in a clear dose–response relationship with both glucocorticoid dosage and treatment duration (7, 8). Another multicenter retrospective study demonstrated that glucocorticoid therapy was the only immunosuppressant associated with 90-day PJP mortality when prednisone dose was ≥10 mg/day (4). It is worth noting that a recent multicenter, double-blind randomized controlled trial highlighted that the benefits and risks of steroids in the treatment of non-HIV PJP may differ from the traditional paradigm from HIV cohorts (9). There is a study suggest that patients with solid tumors, particularly those receiving immunomodulatory therapy, have become an important emerging population for non-HIV-PJP (3). Unlike traditional reports focusing on non-HIV PJP cases involving hematologic or organ transplant patients, this case presents a process where CIP developed after immunotherapy, followed by secondary opportunistic infection with *P. jirovecii* due to corticosteroid intervention, which differs from previous PJP case reports.

The diagnosis of non-HIV PJP remains challenging. Traditional methods such as BAL fluid smear, immunofluorescence, or PCR detection are often limited by sample quality, detection window, and quantitative thresholds in clinical practice (10–12). In contrast, metagenomic next-generation sequencing (mNGS) demonstrates higher pathogen detection rates and broader diagnostic capabilities in critically ill patients. Multiple studies have confirmed that mNGS demonstrates significantly higher diagnostic positivity rates than traditional culture methods in ICU patients with severe pneumonia, and can facilitate early precision antimicrobial therapy (11, 13, 14). Relevant studies indicate that the 30-day and 90-day mortality rates of PJP in non-HIV immunosuppressed patients are 52 and 67%, respectively, which are higher than those in HIV-infected individuals. This suggests that patients with severe PJP urgently require rapid and accurate diagnosis (15, 16). In this case, BALF mNGS identified *P. jirovecii* as the predominant pathogen while simultaneously detecting bacterial and fungal organisms, suggesting that the patient had a co-infection of multiple pathogens rather than a single pathogen infection. This has direct implications for the early application of anti-infective strategies.

A multicenter randomized controlled trial indicated that non-HIV-PJP progresses to severe disease, it often manifests as acute onset, severe hypoxemia, high demand for mechanical ventilation, and poor outcomes (17). Compared to HIV-positive patients, the diagnosis of non-HIV patients is often delayed, leading to further deterioration of the condition (18). During the COVID-19 pandemic, some immunocompromised patients also developed PJP infection. Its clinical manifestations are atypical and progress rapidly, and it is easy to be misdiagnosed (19). A multicenter ICU study demonstrated that the case fatality rate of PJP in non-HIV immunocompromised populations exceeds 50%, and is associated with the need for mechanical ventilation (15). However, there is a lack of case reports in the existing literature regarding the details of respiratory support in the ICU. This case shows in detail the process of lung recruitment and PEEP titration based on respiratory mechanics monitoring which provides a practical path for severe PJP.

As a selective pulmonary vasodilator, iNO can improve oxygenation in the short term by enhancing ventilation-perfusion matching in the lungs (20). It selectively dilates pulmonary vessels in well-ventilated alveoli, reducing intrapulmonary shunt and improving oxygenation. It may also lower pulmonary artery pressure and right ventricular afterload in ARDS-like physiology, supporting cardiopulmonary function (21). A case report on ECMO demonstrated that iNO improved right ventricular function and enhanced oxygenation levels, thereby reducing ECMO duration. iNO achieves these physiological effects by reducing selective pulmonary vascular resistance, optimizing ventilation-perfusion balance, and redistributing blood flow in areas of hypercapnia (22). Although iNO is recommended 24–72 h after onset, studies have shown that it is effective when administered early (23). Regarding the application of iNO in severe pneumonia, existing evidence primarily originates from case reports, with improvements typically confined to short-term oxygenation rather than definitive survival benefit (24). In the present case, inhaled nitric oxide (iNO) was administered following lung recruitment maneuvers and PEEP titration, resulting in a sustained improvement in the oxygenation index. These findings suggest that, in severe PJP, iNO may be better positioned as an adjunctive component of a comprehensive respiratory management strategy rather than as a stand-alone therapeutic intervention.

Importantly, worsening respiratory failure in patients treated for CIP should not be attributed to immune-mediated pneumonitis alone; secondary opportunistic infections must be actively excluded, particularly under prolonged corticosteroid exposure. This case highlights the diagnostic challenge and management complexity of *Pneumocystis jirovecii pneumonia* in patients receiving prolonged high-dose corticosteroids. The combination of mNGS-guided pathogen identification and individualized ICU respiratory support, including lung recruitment, PEEP titration, and adjunctive inhaled nitric oxide, may contribute to favorable outcomes in selected critically ill patients. Clinicians should maintain heightened awareness of opportunistic infections such as PJP in patients who experience respiratory deterioration during or after corticosteroid therapy.

## Limitations

This report has several limitations. First, as a single-case report, the observations cannot establish causality or generalizability regarding the efficacy of lung recruitment manoeuvres, individualized PEEP titration, or adjunctive iNO in severe non-HIV PJP. Second, although BALF mNGS enabled rapid pathogen detection, distinguishing true infection from colonization remains challenging and requires clinical correlation. Further prospective studies are needed to define optimal diagnostic and respiratory support strategies in this population.

## Data Availability

The original contributions presented in the study are included in the article/supplementary material, further inquiries can be directed to the corresponding authors.
